# A High Sensitivity and Anti-Scaling Surface Plasmon Resonance Sensor for Early Screening of Colorectal Cancer

**DOI:** 10.3390/bios16080418

**Published:** 2026-08-03

**Authors:** Ting Jou Ding, Liyuan Wang, Tianyi Chen, Tao Yu, Ching-Jung Chen, Jen-Tsai Liu

**Affiliations:** 1 Department of Biomedical Engineering, Ming Chuan University, Taoyuan City 333321, Taiwan; 2 Research Center for Materials Science and Opti-Electronic Technology, College of Materials Science and Opto-Electronic Technology, University of Chinese Academy of Sciences, Beijing 101408, China; 3 Research Center for Materials Science and Opti-Electronic Technology, School of Electronic, Electrical and Communication Engineering, University of Chinese Academy of Sciences, Beijing 100049, China; 4 Department of General Surgery, Department of Gastrointestinal Surgery, Beijing Hospital, National Center of Gerontology, Institute of Geriatric Medicine, Chinese Academy of Medical Sciences, Beijing 100730, China; 5 Research Center for Materials Science and Opti-Electronic Technology, School of Optoelectronics, University of Chinese Academy of Sciences, Beijing 101408, China

**Keywords:** SPR, colorectal cancer, hemoglobin, antifouling, aptamer, fecal occult blood

## Abstract

Early screening is essential for improving the prognosis of colorectal cancer (CRC), for which fecal occult blood testing (FOBT) remains one of the most widely adopted noninvasive screening approaches. However, conventional FOBT methods often exhibit insufficient sensitivity and limited reliability when handling complex fecal matrices, largely due to nonspecific fouling and cumbersome sample pretreatment procedures. Herein, we report a surface plasmon resonance biosensor integrating a self-assembled anti-fouling interface with DNA aptamer-mediated molecular recognition for sensitive hemoglobin detection in fecal samples. The engineered sensing surface exhibits high interfacial stability and resistance to nonspecific adsorption, enabling direct analysis of fecal samples without complicated pretreatment. The proposed platform achieved a detection limit of 1 nM and a diagnostic accuracy of 97.6%. This study demonstrates the feasibility of SPR-based hemoglobin detection in complex fecal samples using an antifouling sensing interface and highlights its potential as a promising optical biosensing strategy for noninvasive colorectal cancer screening.

## 1. Introduction

Colorectal cancer has a high worldwide incidence. Its morbidity, mortality, standardized incidence rate (SIR), and standardized mortality rate (SMR) all rank highly (top 5) among those of all cancers [1,2,3,4]. The vast majority of rectal cancer cases (approximately 70%) are sporadic [5], and lifestyle, eating habits (drinking), intestinal inflammation [6], and obesity [7] are common influencing factors. The incidence and death rates related to colorectal cancer vary significantly in different countries around the world [8]. With limited advanced medical technology and resources, early diagnosis, early treatment and rectal cancer screening are important strategies for improving the survival rates of colorectal cancer in economically underdeveloped countries or regions [9,10]. The fecal occult blood test (FOBT) is a simple, economical and painless method for colorectal cancer screening. It detects early colorectal cancers and precancerous lesions. It is easy to apply, inexpensive, effective and fast, and it saves human and medical resources. It is suitable for large-scale screening and is the preferred colorectal cancer screening method recommended by domestic and international guidelines [11]. However, the existing chemical fecal occult blood test (gFOBT) and immuno fecal occult blood test (iFOBT) have relatively low sensitivities and are prone to false-positive and false-negative diagnoses [12,13].

**Figure 1:**
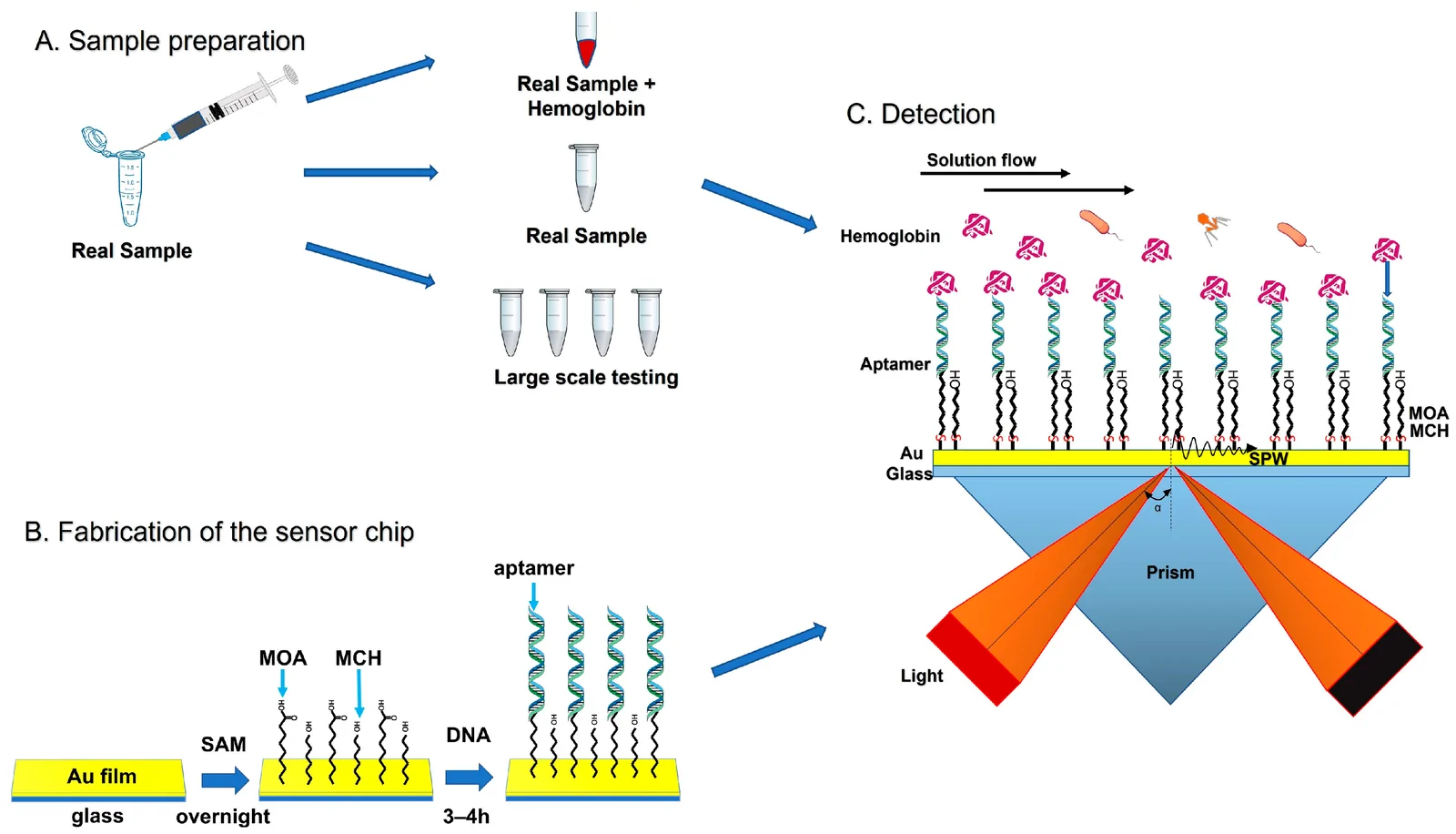
Schematic illustration of the SPR biosensor for fecal occult blood detection in complex fecal samples.

Surface plasmon resonance (SPR) is an optical detection technology. Due to the existence of metal surface plasma waves, the SPR technique reflects the characteristics of samples near the metal film based on the resonance conditions of the metal surface plasma waves. This is a fast, real-time, noninvasive, label-free and highly sensitive detection technology. The SPR technique can be used to measure sample concentrations, film thicknesses, temperatures, and kinetic parameters of intermolecular interactions [14,15]. At present, SPR technology is widely used in medicine [16,17], food safety [18], drug manufacturing [19], environmental monitoring [20], biotechnology [21,22] and many other fields. In this study, we employed self-assembled monolayer technology to immobilize the Hb aptamer onto a self-designed SPR chip for sensitive hemoglobin detection in real fecal matrices. The resulting sensing interface exhibited excellent antifouling performance, effectively minimizing nonspecific adsorption from the complex fecal matrix while maintaining high analytical sensitivity. The proposed biosensor achieved a detection limit as low as 1 nM and demonstrated an overall detection accuracy of 97.6% in real sample analysis. The detailed experimental procedure is illustrated in Figure 1. To the best of our knowledge, this work represents one of the first SPR biosensors developed for hemoglobin detection in real fecal matrices.

## 2. Materials and Methods

### 2.1. Materials and Apparatus

The aptamer was synthesized by Sangon Biotech. Ltd. (Shanghai, China). Bovine serum albumin (65,000 kDa for calculation) (BSA) was purchased from Sigama-Aldrich (Shanghai) Trading Co., Ltd. (Shanghai, China). Human serum albumin protein (65 kDa for calculation) (HSA) was purchased from Shanghai Aladdin Biochemical Technology Co., Ltd. (Shanghai, China). Fetal bovine serum (S601S-500) was purchased from SeraPro (Germany). GIBCO trypsin was purchased from Thermo Fisher Scientific Co., Ltd. (Shanghai, China). 8-Mercaptooctanoic acid (MOA) was purchased from Sigama-Aldrich (Shanghai) Trading Co., Ltd. (Shanghai, China). 6-Mercapto-1-hexanol (MCH), N-Hydroxysulfosuccinimide sodium salt, (sufo NHS) and 1-(3-Dimethylaminopropyl)-3-ethylcarbodiimide hydrochloride (EDC) were purchased from Shanghai Aladdin Biochemical Technology Co., Ltd. (Shanghai, China). Anhydrous ethanol was purchased from Beijing Chemical (Beijing, China). Morpholine ethanesulfonic acid (MES) was purchased from Shanghai Maclean Biochemical Technology Co., Ltd. (Shanghai, China). Deionized water obtained from the Millipore Direct-Q 5UV water purification system was used in all experiments. The refractive index matching oil (E = 1.520) series was purchased from Cargille Laboratories (Cedar Grove, NJ, USA). The buffer solution used in this study was 0.01 M phosphate-buffered solution (PBS) with pH 7.4, which was purchased from Beijing Solarbio Science & Technology Co., Ltd. (Beijing, China). SPR measurements were performed using an MP-SPR Navi™ 210A VASA instrument (BioNavis, Tampere, Finland) for real-time monitoring of resonance angle changes during target detection. The injection, washing, and signal acquisition processes were automatically controlled by the instrument system. A flow-through configuration was used, and the resonance angle shifts were recorded in real time during Hb detection.

### 2.2. Fabrication of the Sensor Chip

Fabrication of the sensor chips was divided into two parts: generation of the surface self-assembled monolayers (SAMs) and grafting of the aptamers [23]. First, the cleaned gold sheet was placed into a prepared MCH: MOA solution with a concentration ratio of 5:1. Due to the formation of Au-S bonds, MOA and MCH were firmly adhered to the surface of the gold film. Then, the chip was placed in a solution with an EDC: NHS concentration ratio of 1:1, and finally, the aptamer was added for grafting. The aptamer used was specifically designed to capture Hb, with a sequence of 5′- GGC AGG AAG ACA AAC ACC AGG TGA GGG AGA CGA CGC GAG TGT TAG GTA GCT GGT CTG TGG TGC TGT-3′ [24]. Due to the presence of many -COOH moieties on the back surface of the SAM, which reacted with the -NH groups on the DNA, the aptamer was firmly fixed to the chip surface. The purpose of mixing MCH was to introduce short-chain -OH groups, which filled gaps in the long chains, ensured that the long chains grew evenly and quantitatively, and improved the success rate and effect of the modification. In addition, the -OH groups neutralized the -COOH groups that had not been grafted with the DNA to ensure the electrical neutrality of the interface. All information on the reagents used is presented in the Supplementary Materials.

### 2.3. Surface Feature Analysis

We characterized the gold film using techniques such as water contact angle measurement, ellipsometry, infrared spectroscopy, X-ray photoelectron spectroscopy (XPS), and SPR air resonance angle, and calculated the surface coverage of the gold film. The water contact angle directly reflects changes in the physical and chemical properties of the interface through changes in the interfacial hydrophilicity. An ellipsometer is a good tool for measuring the film thickness, which directly reflects the growth of the film. In addition, the effect of the air resonance angle on SPR reflects changes in the interfacial film thickness. Infrared spectroscopy can provide information about the chemical composition and functional groups present on the surface of the gold film. It can detect the presence of specific chemical bonds, such as C-O, O-H, or other functional groups, which can indicate the presence of adsorbed species or contaminants on the gold surface. We also used X-ray photoelectron spectroscopy (XPS) to analyze the groups and element ratios on the surfaces of gold films treated by different methods [24,25,26]. Finally, by analyzing the film composition through infrared spectroscopy, the success of the modification was verified. Another factor used to evaluate the modification effect is the surface coverage. The surface coverage P can be expressed as

(1) $$P=\frac{m\times n}{x\times y}$$

where *m* is the number of chains, *n* is the length of a single chain, and *x* and *y* are the length and width of the chip, respectively. In the simulation, the nearest-neighbor interactions between the chain and the surface are considered, and the first unit of each chain always contacts the surface and does not fall off [27,28].

### 2.4. Antifouling Properties

Because fecal samples are highly complex biological matrices, the SPR chip should exhibit excellent anti-scaling performance to minimize nonspecific adsorption and ensure the specific detection of Hb. To test the anti-scaling properties of the surface, proteins, macromolecules and complex solutions were introduced. Nonspecific protein adsorption may be caused by hydrogen bonding, electrostatic, charge transfer and hydrophobic interactions [25]. BSA and HSA were introduced, which contain hydrophobic, hydrophilic, cationic and anionic regions, to test the anti-fouling performance [24,26,29]. In addition, trypsin was selected as a representative biomacromolecule. We used serum diluted 100 times to simulate the complex environment. The three parts were used to confirm whether the modified chip surface accurately detected Hb in a complex environment. A Hb solution with a concentration of 50 nM, HSA and BSA solutions with concentrations of 500 μM, a trypsin solution with a concentration of 25 mg/mL and serum diluted 100 times were used. A 0.01 M PBS with a pH of 7.4 was used as the buffer solution. The serum was also diluted with the PBS, and a new SPR chip was used for each experiment. First, the PBS was introduced to determine the baseline, then the sample to be tested was introduced, and finally, the PBS was reintroduced to clean the SPR chip surface. After the final cleaning, only the substances firmly adsorbed on the surface were left, and the other substances were washed away. The final resonance angle shift (ΔRU) after signal stabilization was used to evaluate the anti-fouling performance of the modified sensor surface.

### 2.5. Hb Detection

A 0.01 M PBS (pH 7.4) was used as the detection buffer to mimic physiological conditions. Different concentrations of hemoglobin (Hb) were prepared in PBS and introduced into the SPR sensing system to establish the calibration curve. Due to the slower metabolism of hemoglobin at low temperatures and the longer survival time [30,31,32], all solutions to be tested were stored in an ice bath during the experiment to prevent Hb denaturation caused by high temperatures.

### 2.6. Real Sample Testing

Real fecal sample analysis was performed using human fecal matrices, including blank fecal samples and hemoglobin (Hb)-spiked fecal samples, to evaluate the applicability of the proposed SPR biosensor in complex fecal environments. Real samples were collected from a volunteer and stored at −20 °C before analysis. The collection and use of real samples in this study were approved by the Ethics Committee of Beijing Hospital (Approval No. 2025BJYYEC-KY106-01). Prior to the experiments, the samples were equilibrated at 5 °C and diluted with PBS buffer to prepare fecal diluents. The analysis of real fecal samples was divided into two parts. First, standard experiments were conducted with feces that did not contain hemoglobin. Second, appropriate amounts of human Hb were added to the filtered fecal diluents to prepare Hb-spiked fecal samples, and the corresponding SPR angle shifts were sequentially recorded. We used many stool samples containing different amounts of hemoglobin to determine the accuracy of the system.

For performance comparison, commercially available fecal occult blood test kits representing widely used fecal occult blood testing methods were also employed. Two immunogold-based test kits, namely the ABON Fecal Occult Blood (FOB) Test Kit (colloidal gold method) (ABON Biopharm (Hangzhou) Co., Ltd., Hangzhou, China) and the Deep Blue Fecal Occult Blood Immunoassay Kit (Anhui Deep Blue Medical Technology Co., Ltd., Anhui, China), were used. Commercial o-toluidine and pyramidon fecal occult blood test kits (Solarbio Science & Technology Co., Ltd., Beijing, China) were also included. Fifty independent hemoglobin-spiked human fecal samples were tested for each commercial method, and the corresponding qualitative detection accuracies were compared with those of the proposed SPR biosensor.

## 3. Results and Discussion

### 3.1. Surface Feature Analyses

**Figure 2:**
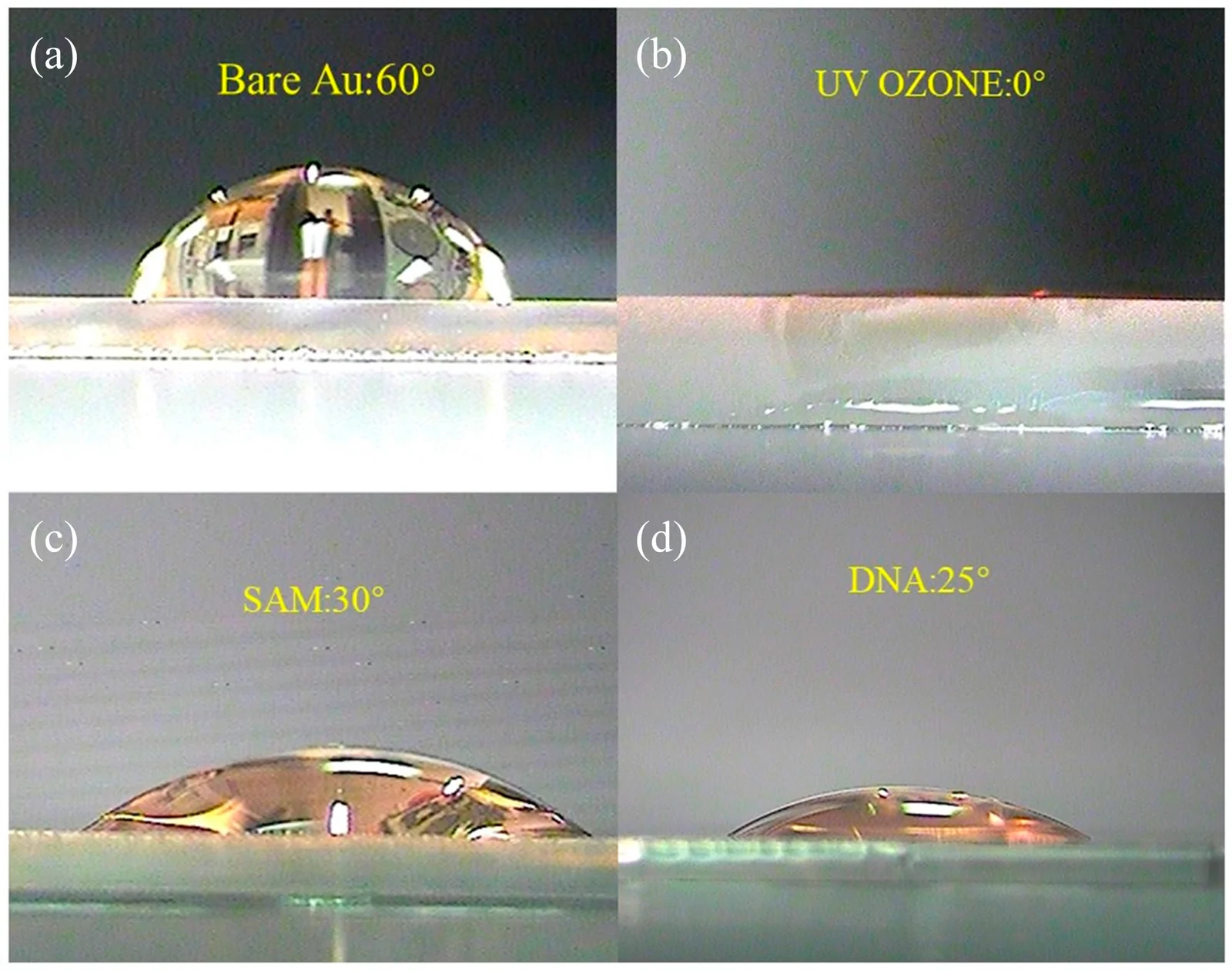
Evolution of water contact angles during stepwise surface functionalization of the SPR sensing interface. (**a**) Bare Au chip, (**b**) ozone-cleaned Au chip, (**c**) SAM-modified Au chip, (**d**) aptamer-functionalized Au chip.

The DI water contact angle for bare gold is generally above 60° (Figure 2a). However, after ozone cleaning, the surface energy of Au increases, and its affinity for water is enhanced. Therefore, the water contact angle on the surface of a completely clean SPR chip is very small, almost 0 (Figure 2b) [33]. The contact angle between DI and water increased to approximately 30° (Figure 2c) after SAM formation. This is because both carboxyl and hydroxyl groups are hydrophilic [34] and carboxyl groups are more hydrophilic than hydroxyl groups. The higher the surface activity is, the better the SAM effect. After grafting the aptamer, the hydrophilic phosphate groups reduced the contact angle between the surface and water to approximately 25° (Figure 2d) [35].

In addition, SPR is a powerful tool for observing interfacial phenomena, and it easily determines the thicknesses of thin films on the interface [36]. In this study, the air resonance angles of the Au chips, SAM chips, and aptamer-grafted chips were measured by SPR, which resulted in resonance angles of 43.43°, 43.56°, and 43.75°, respectively. Ellipsometers are used to measure the thicknesses of thin films with known optical parameters [37]. Therefore, an ellipsometer was used to measure the average film thicknesses of the bare gold, SAM chips, and aptamer-grafted chips. The film thicknesses were 50 nm, 54.88 nm, and 62.184 nm, respectively. This was due to the growth of self-assembled monolayers on the surface of the gold film after SAM and the addition of a uniform aptamer molecular layer after grafting. Based on the thicknesses measured with the ellipsometer and the air resonance angles measured by SPR, it was concluded that the thickness of the self-assembled monolayer was smaller than that of the aptamer layer. These data, coupled with changes in the water contact angle, confirmed the success of the transformations.

The reflectivity diagrams for the different gold sheets are shown in Figure S1. As seen from the figure, the reflectivity of bare gold itself was approximately 100% because the background noise was also caused by bare gold. The reflectivities of less than 100% may have been due to subtle differences between the different bare gold sheets, as well as interference from the coating methods and indoor natural light. Compared with the bare gold spectrum, the spectrum obtained after mixed SAM modification exhibited characteristic absorption bands corresponding to the functional groups of both MCH and MOA, confirming the successful formation of the mixed self-assembled monolayer on the gold surface. The presence of both hydroxyl (-OH) and carboxyl (-COOH) functional groups indicates that both MCH and MOA were successfully introduced onto the modified gold surface.

**Figure 3:**
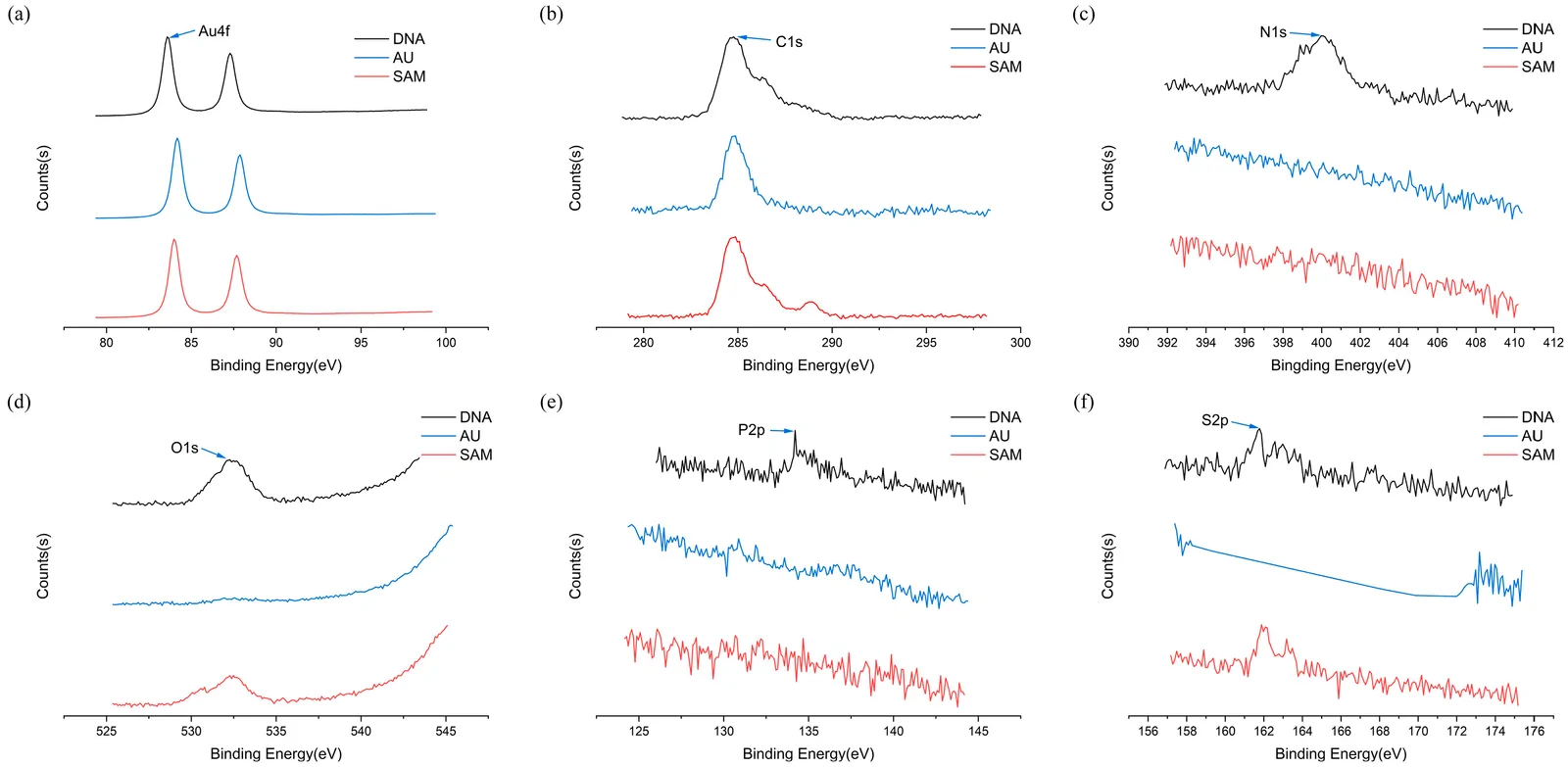
XPS spectra of bare gold corresponding to six elements: (**a**) Au4f, (**b**) C1s, (**c**) N1s, (**d**) O1s, (**e**) P2p, and (**f**) S2p, the gold sheet after mixed solution SAM and the gold sheet after DNA aptamer grafting.

We conducted XPS studies on a gold sheet cleaned with ozone, a gold sheet treated with the MOA and MCH mixed solution, and a gold sheet grafted with DNA aptamers. The tested elements were Au, C, O, S, P, and N, and the energy spectra are shown in Figure 3. Figure 3b,d,f show that the bare gold surface contained no other elements, and the trace amounts of C and O were due to the high surface activity of the Au after ozone cleaning; the surface adsorbed oil and pollutants from the air during the test process, which resulted in additional peaks. After the SAM treatment, the XPS peaks for C, O and S were obvious, proving that MOA and MCH were successfully assembled on the surface. Figure 3c,e show that there were no N and P signals on the SAM-containing surface or the bare gold, while the gold sheet showed obvious peaks after DNA grafting, which were mainly due to the N and P moieties in the DNA, proving that the DNA was successfully grafted onto the MOA chains.

### 3.2. Surface Coverage Analysis

It was assumed that the coverage of MOA molecules on the gold film surface was 100% when MOA was used for a single SAM process, and it was similarly assumed that the coverage of MCH molecules on the gold film surface was 100% when MCH was used for a single SAM process. Then, the approximate coverage of the mixed SAM surface was calculated. First, the contact angle was used for estimation. Since there was a linear relationship between the contact angle and surface coverage, and it decreased with increasing coverage [38,39], the coverage was calculated with the data in Table S2. The water contact angle for a monolayer of MOA was 28°, and the water contact angle for a monolayer of MCH was 25°. The specific coverage was calculated with Formula (2):

(2) $$p=\frac{D_{Au}-D}{C_{MOA}\left(D_{Au}-D_{MOA}\right)+C_{MCH}\left(D_{Au}-D_{MCH}\right)}$$

where *p* is the coverage of the surface, *D_Au_* is the water contact angle of bare gold (60°), *D* is the water contact angle of the surface to be measured, *D_MOA_* and *D_MCH_* are the water contact angles of the MOA surface and MCH surface with 100% coverage, respectively, and *C_MOA_* and *C_MCH_* are the relative concentrations of MOA and MCH, respectively. In this solution, the concentration ratio of MOA to MCH was 1:5. In addition, the water contact angles of the MOA surface and MCH surface with 100% coverage were obtained from Table S2. After calculation, the coverage p of the mixed SAM surface was approximately 87%.

In addition, based on the average thickness, if the MOA molecules completely covered the surface, the average film thickness would be 56.36 nm. After subtracting the original gold film thickness of 50 nm, the thickness of the MOA monolayer should be 6.36 nm. For the MCH monolayer, the thickness was 5.16 nm. The specific coverage was calculated with Formula (3):

(3) $$p=\frac{T}{C_{MOA}\times T_{MOA}+C_{MCH}\times T_{MCH}}$$

*C_MOA_* and *C_MCH_* still represent the relative concentrations of MOA and MCH, *T_MOA_* and *T_MCH_* are the film thicknesses of the pure MOA monolayer and pure MCH monolayer, and *T* is the film thickness of the surface to be measured. The calculated coverage p of the mixed SAM surface was approximately 91%.

The C, O and S XPS peaks for the gold sheet treated with the mixed SAM solution containing MOA and MCH are shown in Figure S2. Figure S2a shows that the main C groups formed were C-C, C-OH and O=C-O, which corresponded to the three peaks at 284.8 eV, 286.4 eV and 288.9 eV, respectively. Figure S2b shows that the main groups formed for O were C-OH, O=C-O and C-O, which corresponded to the peaks at 532.2 eV, 533.6 eV and 530.5 eV, respectively. The C-OH signal is attributed to the hydroxyl (-OH) groups of MCH, whereas the O=C-O signal originates from the carboxyl (-COOH) groups of MOA, confirming the successful introduction of both functional molecules onto the gold surface. Figure S2c shows the S 2p spectrum, in which the peak at 161.9 eV is assigned to Au-S bonds, indicating the successful chemisorption of the thiol molecules onto the gold surface during SAM formation. The weak peak at 165.5 eV is assigned to oxidized sulfur species (S-O), indicating that only a small fraction of the sulfur atoms underwent oxidation during the modification process. These results collectively confirm the successful formation of the mixed self-assembled monolayer on the gold surface.

The C, N, O and P XPS spectra are shown in Figure S3 for a gold sheet grafted with DNA aptamers. Among them, the C (Figure S3a) and O (Figure S3c) spectra were not significantly different from those seen after SAM formation. Figure S3b shows that the N elements were mainly contained in amine (-NH_2_) and amide (O=C-N-H) groups. They gave the peaks at 399.6 eV and 400.5 eV, respectively. This was mainly due to the amide groups formed by dehydration of the amino groups in the DNA and the carboxyl groups onto the ends of the MOA molecules. The amide groups ensured that the DNA aptamer could be grafted at the ends of the MOA chains. The peak at 133.3 eV corresponded to P-O moieties in the P XPS spectrum shown in Figure S3d, which were present in the phosphates of the DNA.

In Table 1, it was assumed that the entire Au film comprised 100 Au atoms, while the mixed SAM layer contained approximately 10% grafted mercapto groups (estimated from the Au:S ratio from the XPS results of the second stage). For the third stage, the sulfhydryl-based DNA grafting ratio was approximately 6.7% (the P:S ratio for the third stage was estimated to be approximately 70 phosphates (P) per grafted DNA, and one DNA corresponded to 6 sulfhydryl groups (MCH:MOA = 5:1)). That is, if all sulfhydryl groups were connected, the P:S ratio would be 70:6. According to the elemental contents listed in the table, the XPS data for the fitness stage gave a ratio of 1.71:2.2. Therefore, in the first layer of Au atoms, approximately 0.67% of the DNA was grafted onto the Au atoms.

If the entire surface of the gold film was Au, the grafting ratio of the mixed SAM layer could be obtained from the ratio of Au to S at the SAM stage, which was approximately 11%. The grafting rate of the DNA aptamer was obtained from the ratio of N and S after DNA grafting. Because only MOA molecules in the mixed SAM layer were grafted with DNA, as shown in Figure S3b, we knew that the proportion of MOA molecules was approximately 2/3, and each base contained one N. The total number of 72 base pairs in the aptamer we used should contain 144 N atoms plus 145 N atoms for the chain end-modified NH_2_ groups. Therefore, if the SAM was full of aptamers, the N:S ratio should be 145:1.5. The actual ratio was 7.83:2.2, so the proportion of DNA grafted onto the SAM surface was 3.7%.

The numbers of MOA and MCH chains needed for coverage were 8 and 7, respectively, so these values were brought into formula [40]. For convenient calculations, we assumed that there were 10 × 10 or 100 gold atoms on the gold sheet, that is, *x* = *y* = 10, *m* = 11, and the average chain length was 7.5, so the surface coverage of the mixed SAM layer was 82.5%, which was similar to the proportions of the other elements after the SAM process except for Au. This was close to 87% and 91% of the average film thickness calculated from the contact angles.

### 3.3. Antifouling Properties

**Figure 4:**
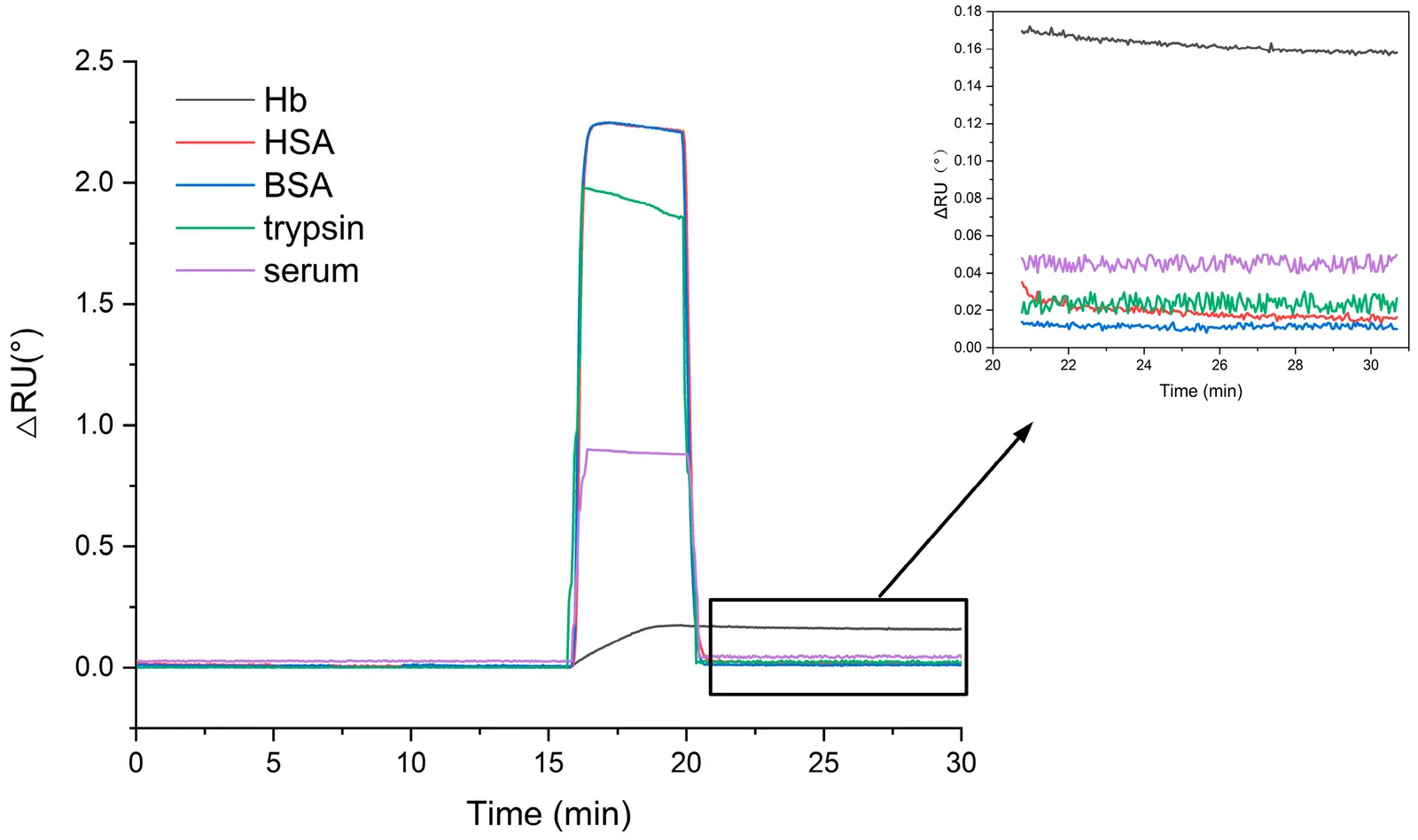
SPR responses of Hb, BSA, HSA, trypsin and serum.

To reduce the differences caused by the properties of the SPR chips, we used the relative SPR values to detect specific adsorption. As shown in Figure 4, a stable baseline was obtained after flowing PBS solution for 15 min. When the sample solution to be tested was injected onto the SPR chip, the SPR angle response was rapid; it rose rapidly, then fell slowly, and finally reached equilibrium. This process took approximately 5 min, and then, after 20 min, the PBS was injected again to clean the chip surface. The SPR angle dropped rapidly. Because the concentrations of other solutions were much higher than that of the Hb solution, the increase should have been more significant. After injecting the PBS again for cleaning, molecules that were not specifically adsorbed did not remain firmly on the chip surface, so the SPR angle decreased almost to the baseline. However, due to specific adsorption, the Hb was firmly adsorbed on the interface, so the SPR angle after cleaning remained higher than the baseline. It should be noted that the SPR angle for the serum after cleaning was also slightly higher than the baseline. This occurred because during the preparation process, the Hb in the serum was not completely separated, and there was still a small amount of Hb in the serum. This Hb was also specifically adsorbed, but due to the small amount of Hb, the equilibrium curve only changed slightly.

As shown in Figure 4, for other solutions with concentrations much higher than that of the Hb solution, the SPR response for the Hb solution was more than 15 times higher than those of other sample solutions. Therefore, the modified chip showed good specificity for Hb and strong anti-scaling ability, which ensured accurate adsorption of the Hb in complex environments and the feasibility of Hb trace analysis.

### 3.4. Experimental Results

**Figure 5:**
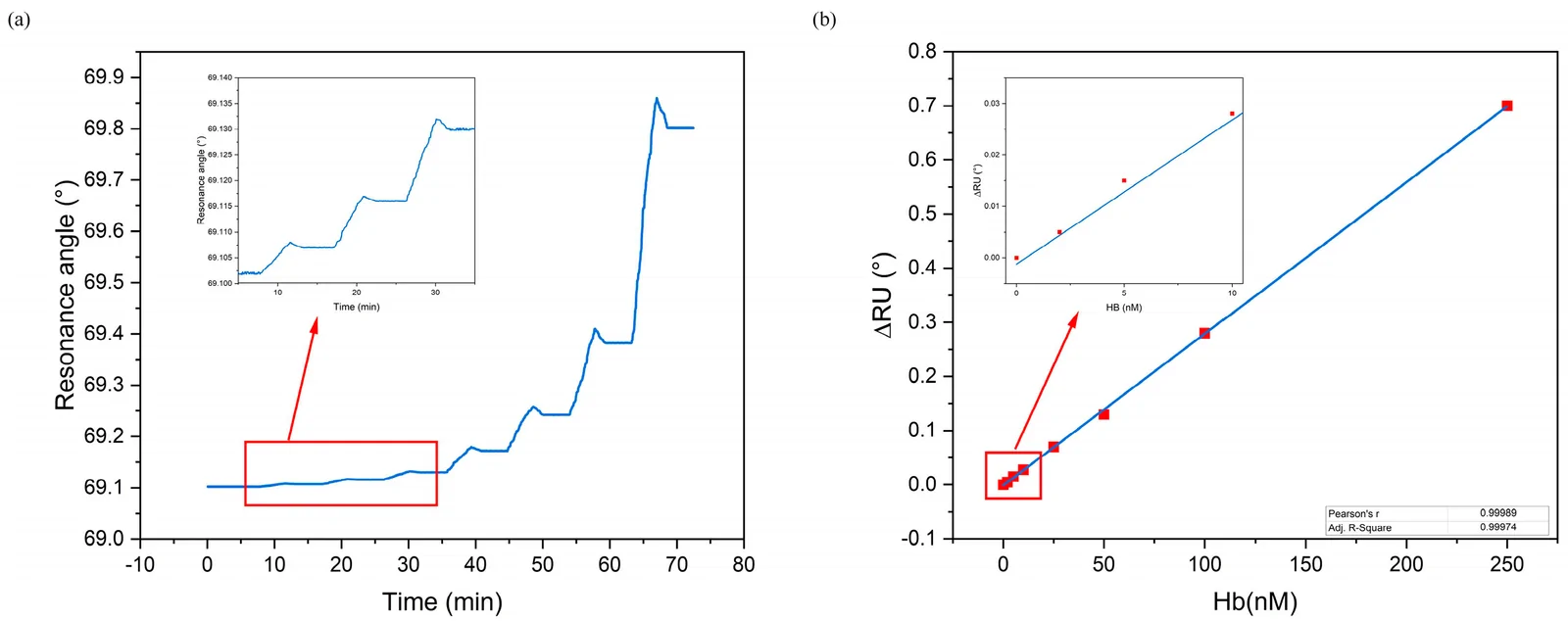
(**a**) Plot of the SPR angle versus time and (**b**) plot of SPR angle versus Hb concentration and the linear fit.

As shown in Figure 5a, after injecting the PBS and stabilizing the data, a baseline for PBS was obtained, which was also the baseline for the entire experiment. Then, Hb solutions with concentrations of 2 nM, 5 nM, 10 nM, 25 nM, 50 nM, 100 nM, and 250 nM were successively introduced to simulate fecal occult blood samples with different concentrations, and a plot of the SPR angle versus time was obtained. After each change to an Hb solution with different concentrations, the SPR angle rose rapidly, reached a peak, slowly declined, and finally reached equilibrium. When equilibrium was finally reached, the SPR angle was significantly greater than the SPR angle seen before the Hb solution was injected. This is because binding of the hemoglobin to the aptamers is a dynamic equilibrium process involving adsorption and desorption. When the Hb solution first flowed into the SPR flow chamber, the increased SPR concentration disrupted the original adsorption–desorption equilibrium, and the adsorption rate was greater than the desorption rate. At this point, adsorption mainly occurred at the interface. In addition, the higher the concentration of the solution, the greater the SPR angle, so at this time, the SPR angle rose rapidly. In this process, although the adsorption rate was greater than the desorption rate, the adsorption rate gradually slowed, and the desorption rate gradually increased as the concentration increased. Therefore, after the adsorption amount reached the maximum value, desorption became the main phenomenon. At this point, the SPR angle slowly decreased. During this process, the rates for adsorption and desorption gradually slowed and then tended to equalize, and equilibrium was reached based on the new sample concentration, i.e., the adsorption and desorption equilibrium.

Since the equilibrium between adsorption and desorption was related to the concentration of the substance, the corresponding equilibrium varied with the Hb concentration. The higher the concentration was, the greater the amount adsorbed and the greater the SPR angle. The equilibrium resonance angle shifts extracted from the SPR sensorgrams were plotted against the corresponding Hb concentrations, as shown in Figure 5b. The linear fit for the plot of SPR angle versus hemoglobin concentration was very good, and the R^2^ value was 0.9998, indicating that the SPR angle was directly and linearly related to the concentration of the detected substance due to the adsorption phenomenon occurring at the interface.

### 3.5. Real Sample Tests

A real sample containing fecal occult blood was diluted with 0.01 M PBS, filtered, passed into the system after the system was stable, and then flushed with the buffer solution after five minutes to obtain the SPR angle change diagram, as shown in Figure S4. The figure shows that when the SPR angle was stable, the solution to be measured was introduced, and the SPR angle immediately rose rapidly and then the plot gradually became flatter. After the surface was washed with the buffer solution, the SPR angle dropped sharply and then remained stable. The resonance angle seen for the final equilibrium state was higher than that of the initial equilibrium state. The sharp rise in the SPR angle seen in the figure was caused by two factors. First, the sample contained hemoglobin, and it combined with the surface DNA aptamers. In addition, the sample environment was complex, and there were many other substances adsorbed on the surface. The sharp decline seen after flushing was due to substances other than Hb in the fecal sample, which were nonspecifically adsorbed onto the surface and washed away by the buffer solution. Therefore, only Hb was firmly bound to the aptamer on the surface after flushing, so the equilibrium resonance angle of the final state was higher than that of the initial state, which proved that the system indeed detected trace Hb in the fecal sample.

**Figure 6:**
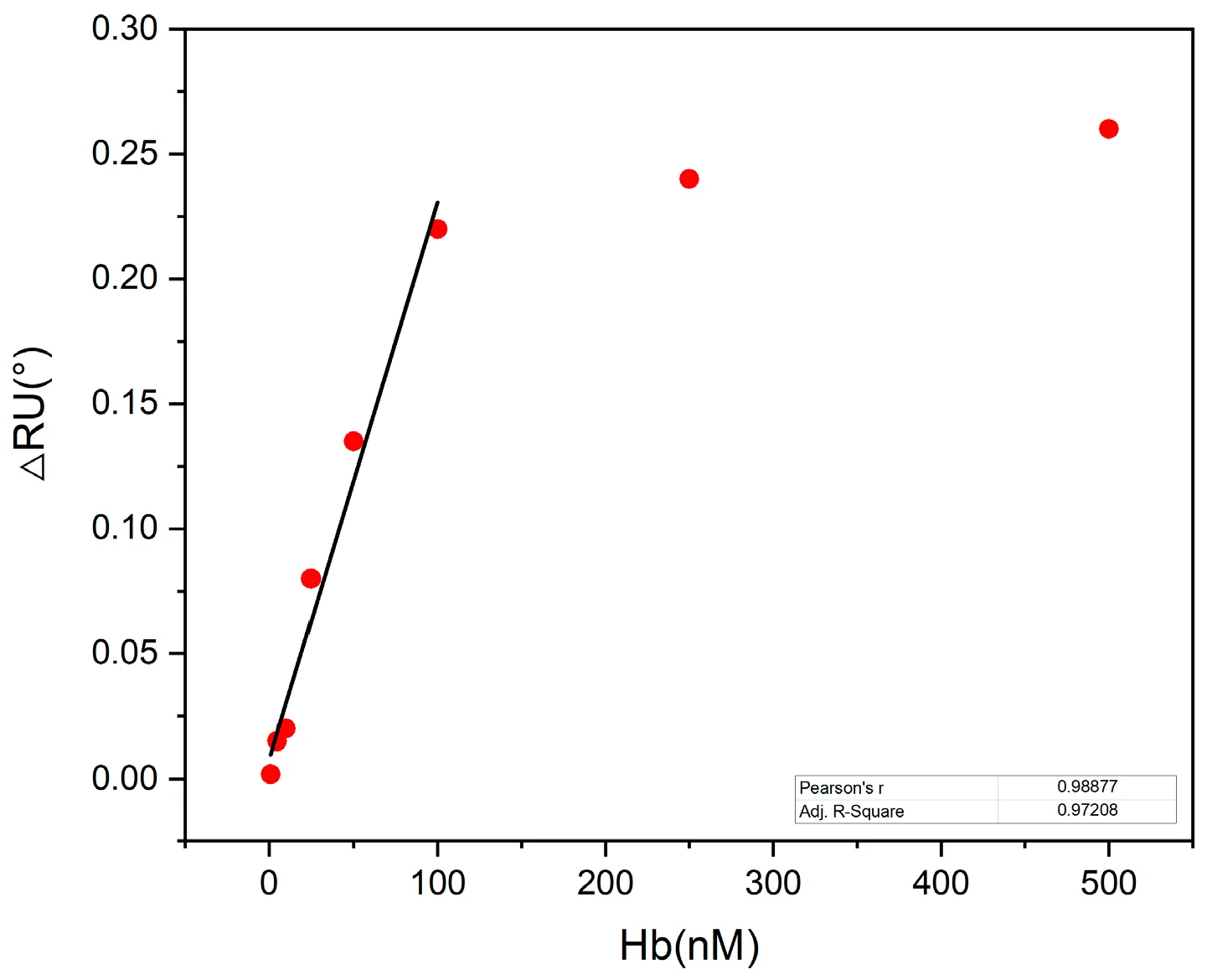
SPR responses of the proposed biosensor toward Hb at different concentrations and the corresponding linear fitting analysis in the linear range.

The fecal sample solution without hemoglobin was diluted and filtered, and then spiked with different concentrations of hemoglobin to simulate fecal occult blood samples with different Hb levels. These samples were introduced into the SPR system, followed by buffer washing, and the corresponding resonance angle shifts were recorded for each concentration. The relationship between the SPR angle shifts and Hb concentration over the range of 1–500 nM is shown in Figure 6. The figure shows the linearity of the plot for fecal occult blood solutions was good for Hb concentrations between 1 nM and 100 nM, but the resonance angle did not change significantly when the concentration increased above 100 nM, perhaps because binding between the aptamer and hemoglobin had reached saturation. Therefore, linear regression was performed using the data points within the range of 1–100 nM, resulting in a great linear fitting relationship with an R2 value of 0.97208. Notably, a hemoglobin concentration of approximately 100 ng/mL (≈1.55 nM) [41], which is a commonly used cut-off value for fecal occult blood screening, falls within this linear detection range. Therefore, the detection capability of the proposed SPR biosensor covers the clinically relevant hemoglobin concentration required for fecal occult blood screening. The narrower linear range observed in fecal samples compared with the standard Hb solution prepared in PBS buffer may be attributed to the more complex fecal matrix, which could affect the accessibility of Hb molecules to the sensing interface.

**Figure 7:**
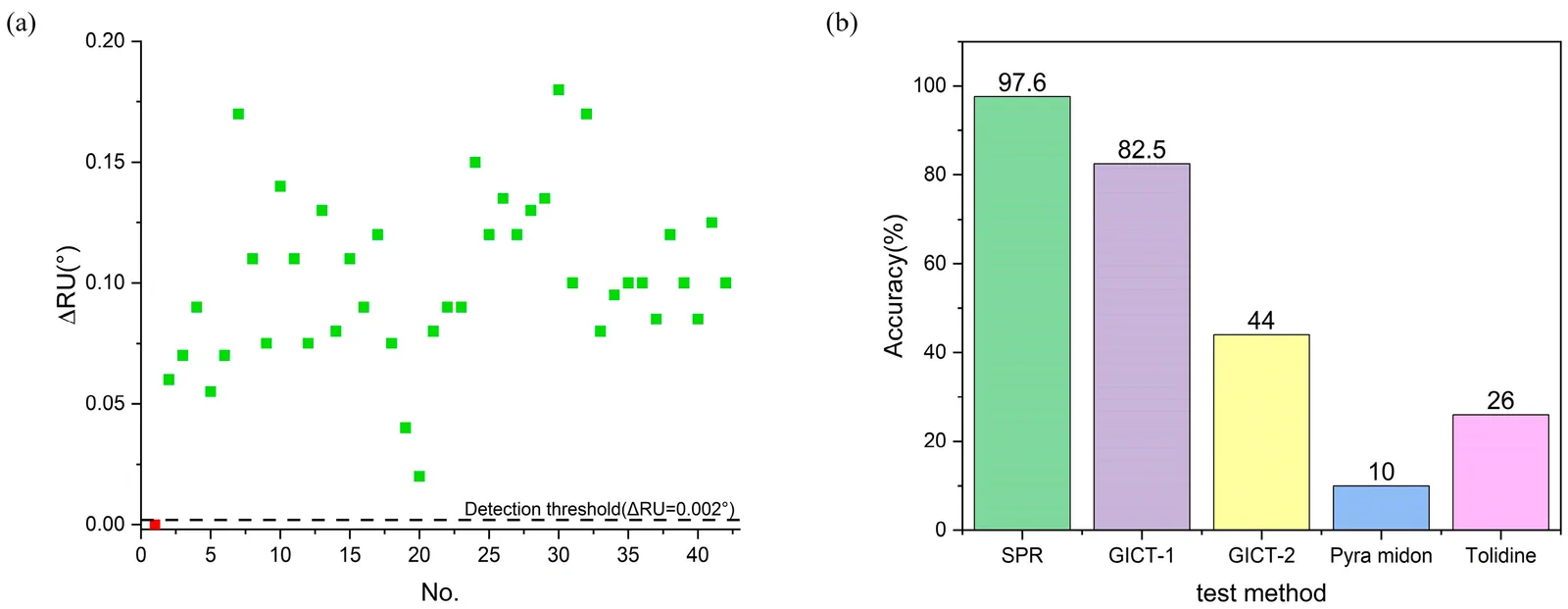
Evaluation of the accuracy of the SPR sensing system. (**a**) Scatter plot of qualitative detection results for 42 real fecal occult blood samples. Red and green dots indicate failed and successful detection, respectively. (**b**) Comparison of the accuracies of different methods for fecal occult blood detection.

Based on the baseline fluctuation of the SPR system, a resonance angle shift exceeding 0.002° was considered a positive result for qualitative fecal occult blood detection. Therefore, 42 hemoglobin-spiked human fecal samples with unknown concentrations were prepared and qualitatively analyzed to test the detection capability of the system. The qualitative detection results are shown in Figure 7a. As indicated by the dashed line (ΔRU = 0.002°), samples with ΔRU values greater than the detection threshold were regarded as successfully detected. Among the 42 hemoglobin-spiked human fecal samples, only one sample was not successfully detected, resulting in an overall detection accuracy of 97.6%.

To further evaluate the performance of the proposed SPR sensing system, several commercially available fecal occult blood test kits representing widely used fecal occult blood testing methods were selected for comparative evaluation, including two immunogold labeling-based methods, as well as the o-toluidine and pyramidon methods. The immunogold labeling method detects hemoglobin through specific antigen–antibody recognition, whereas the o-toluidine and pyramidon methods are conventional chemical assays based on the pseudoperoxidase activity of hemoglobin. Fifty independent tests were conducted for each method. The corresponding accuracies are presented in Figure 7b. The SPR method exhibited the highest accuracy among all tested methods, demonstrating its superior performance for fecal occult blood detection. More importantly, these results demonstrate that the proposed antifouling sensing strategy enables reliable SPR detection of hemoglobin in complex fecal samples, extending the application of SPR biosensing from conventional laboratory measurements to a clinically relevant fecal matrix for fecal occult blood testing.

## 4. Conclusions

In this study, a surface plasmon resonance system was used for the first time to test fecal occult blood. We developed a technology for grafting DNA aptamers onto SPR chips using self-assembled monolayers to detect Hb in real samples, which provided high coverage and antifouling performance. We characterized the entire process of SPR chip fabrication to ensure the successful preparation of the chips, and calculated their surface coverage. The results showed that the surface coverage of the mixed SAM layer was about 87%. We introduced different types of interferents to test the antifouling performance of the chip, ensuring highly specific recognition of hemoglobin in complex samples. The experimental results indicate that the minimum detection limit reached 1 nM and exhibited good linearity. In tests of actual samples, the accuracy rate was as high as 97.6%, which was far superior to that of the other detection methods. This technology confirms the possibility of using SPR technology for fecal occult blood detection and provides a new research direction for low-cost, large-scale early cancer screening in the future.

## Figures and Tables

**Table 1:** Atom percentages of the elements from the XPS spectra.

Modification Process	Au 4f (Atomic %)	S 2p (Atomic %)	C 1s (Atomic %)	O 1s (Atomic %)	N 1s (Atomic %)	P 2p (Atomic %)
Bare Au	61	3.84	26.2	2.9	2.38	3.67
SAM	22.45	2.51	52.82	17.65	1.99	2.57
DNA	17.77	2.2	48.82	21.67	7.83	1.71

## Data Availability

All data supporting the graphical results are contained in the figures and tables within the Supplementary Materials of this article. No external or independent datasets were used in this study.

## Supplementary Materials

The following supporting information can be downloaded at https://www.mdpi.com/article/10.3390/bios16080418/s1, Figure S1. Infrared spectral reflectance of bare gold film after single MOA, single MCH, mixed SAM and grafted DNA aptamer. Figure S2. High-resolution XPS spectra of (1) C 1s, (2) O 1s, and (3) S 2p obtained from the gold chip after self-assembly with the mixed MOA/MCH solution. Figure S3. High-resolution XPS spectra of (a) C 1s, (b) N 1s, (c) O 1s, and (d) P 2p for the gold chip after DNA grafting. Figure S4. Real-time SPR resonance angle response during real-sample detection. Table S1 Changes of SPR chip test data before and after modification. Table S2 Contact angle and film thickness of different SAM surfaces.
