# Supplementary material for: A High Sensitivity and Anti-Scaling Surface Plasmon Resonance Sensor for Early Screening of Colorectal Cancer
Source: Biosensors (Basel). 2026 Aug 3;16(8):418. doi: 10.3390/bios16080418 (PMC13510364; doi:10.3390/bios16080418)
Supplement: Supplementary file 1 [file biosensors-16-00418-s001.zip › biosensors-4421902-supplementary.pdf]

biosensors

MDPI

Supplementary Materials

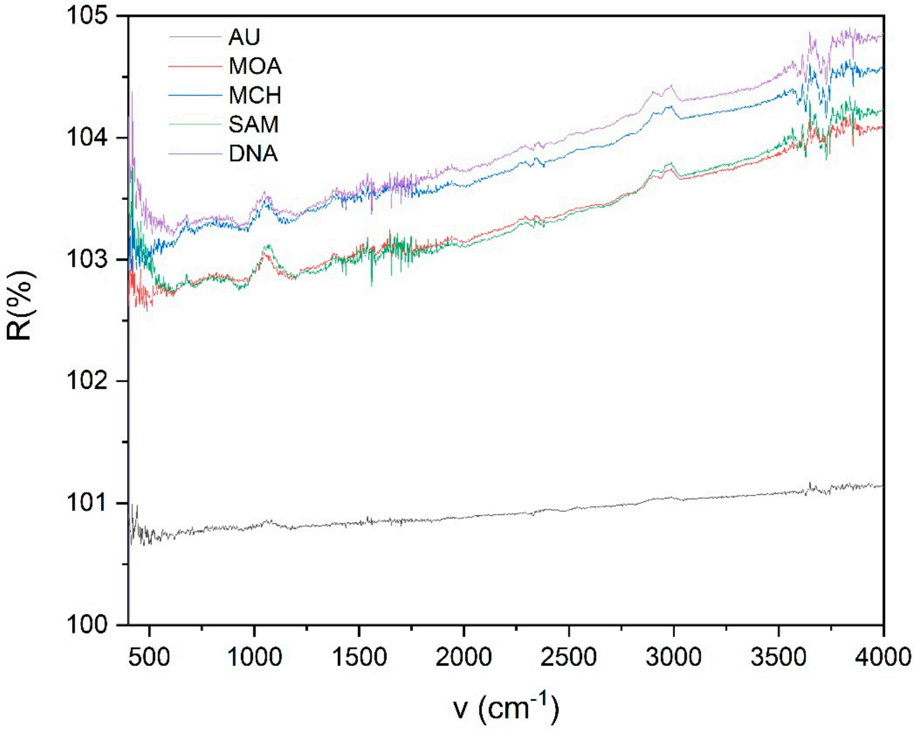

Figure S1. Infrared spectral reflectance of bare gold film after single MOA, single MCH, mixed SAM and grafted DNA aptamer.

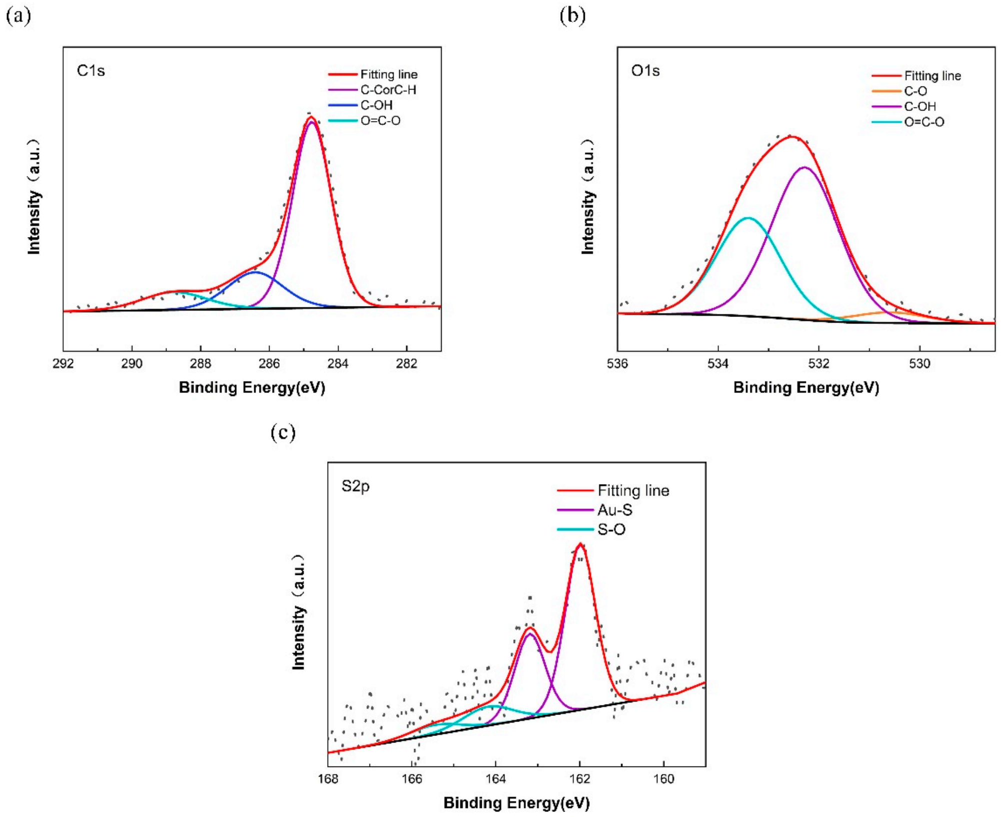

Figure S2. High-resolution XPS spectra of (1) C 1s, (2) O 1s, and (3) S 2p obtained from the gold chip after self-assembly with the mixed MOA/MCH solution.

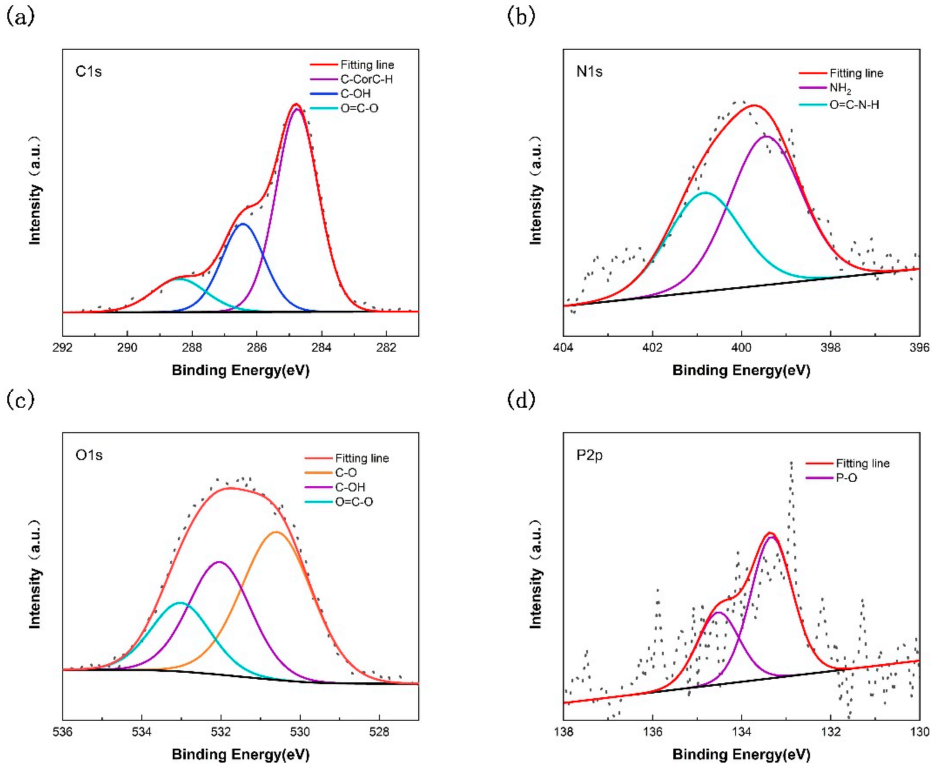

Figure S3. High-resolution XPS spectra of (a) C 1s, (b) N 1s, (c) O 1s, and (d) P 2p for the gold chip after DNA grafting.

biosensors

MDPI

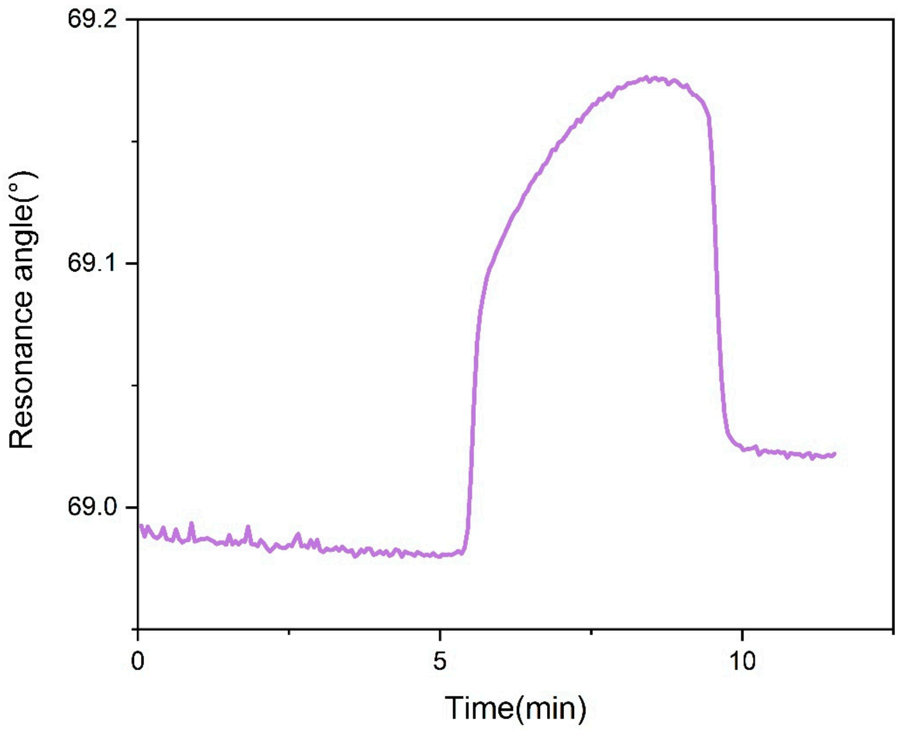

Figure S4. Real-time SPR resonance angle response during real-sample detection.

Table S1 Changes of SPR chip test data before and after modification.

Data	Bare Au	After SAM	After grafting DNA
Contact angle of Water	60°	30°	25°
Air resonance angle	43.43°	43.56°	43.75°
Film thickness	50nm	54.88nm	62.184nm

Table S2 Contact angle and film thickness of different SAM surfaces.

SAM Composition	Contact angle	Average thickness
MOA	28°	56.36nm
MCH	25°	55.16nm
MOA-MCH	30°	54.88nm
